# Microbiological and molecular analysis of the microbiota of insects present in the surgical area

**DOI:** 10.1017/ash.2025.10202

**Published:** 2025-11-24

**Authors:** Christian Cadena-Cruz, Emilse Vásquez Avendaño, Norka Helena Márquez Blanco, Andrea Bolaño Villafañe, Jandro Jose Bolaño Arenas, Carlos Romero Orozco, Juan Reátiga Aguilar, Lisha Maria Cruz Soto, Carlos Mario Moscote Terán, Jose Villarreal-Camacho

**Affiliations:** 1 Universidad Libre Seccional Barranquilla – Programa de Instrumentación Quirúrgica, Barranquilla, Colombia; 2 Universidad Libre Seccional Barranquilla – Programa de Bacteriología, Barranquilla, Colombia; 3 Universidad Libre Seccional Barranquilla – Programa de Medicina, Barranquilla, Colombia; 4 Fundación Clínica Campbell, Barranquilla, Colombia

## Abstract

**Background:**

The presence of insects in hospital environments poses a potential risk for the dissemination of pathogenic bacteria, including multidrug-resistant species. Despite strict sanitation protocols, some arthropod populations persist in less regulated areas, potentially acting as mechanical vectors of bacterial contamination.

**Objectives:**

This study aimed to analyze the bacterial diversity associated with insects collected in hospital settings and assess their potential role in spreading pathogens relevant to public health.

**Methods:**

A descriptive observational approach was employed to identify and classify bacterial taxa associated with hospital-collected insects. High-throughput sequencing was used for taxonomic classification at the phylum, family, and genus levels.

**Results:**

Proteobacteria was the predominant phylum, additionally we found families such as Moraxellaceae and Mycobacteriaceae, known to include clinically relevant species. The genera Acinetobacter and Mycobacterium were particularly abundant in some samples, raising concerns about their potential role in nosocomial infections. Other identified bacteria included Pseudomonas, Escherichia, and Shigella, albeit at lower frequencies. The persistence of these bacteria in hospital environments suggests that insects may contribute to their dissemination.

**Conclusions:**

The findings highlight the need for enhanced arthropod control measures in healthcare facilities as part of routine biosecurity protocols. The presence of multidrug-resistant bacteria associated with hospital-dwelling insects reinforces their role in pathogen transmission, emphasizing the importance of comprehensive vector management strategies to mitigate nosocomial infection risks.

## Introduction

Healthcare-Associated Infections (HAIs) are classified as adverse events arising from medical care that unintentionally result in patient harm. These infections can affect the respiratory and urinary tracts, skin, soft tissues, gastrointestinal tract, and mucous membranes. The transmission routes involve patient contact with surfaces, food, water, air, medical devices, and healthcare personnel.^1^ This issue is further exacerbated by the presence of multidrug-resistant pathogens, which complicate patient prognosis and recovery.^2^

The surveillance and reporting of HAIs are of significant interest to healthcare systems worldwide, as they facilitate the implementation of monitoring, detection, control, and management programs, as recommended by the World Health Organization (WHO).^3^ In Colombia, the Ministry of Health established a program in 2018 with these objectives.^4^ However, existing databases do not explicitly associate the presence or movement of arthropods with the incidence of HAIs in hospital settings. Nevertheless, the microbiota and the corresponding microbiome carried by these organisms include a repertoire of pathogens, some of which have been documented with multidrug-resistant phenotypes.^5–12^ This could contribute to increased morbidity and mortality rates, deficiencies in epidemiological surveillance systems, and inadequate control of these microbial vectors.^13^

This study investigates the relationship between the microbiota present in arthropods collected from the surgical area of a healthcare facility and the presence of potentially pathogenic bacteria. Through microbial composition analysis of these invertebrates, employing next-generation sequencing (NGS) of the 16S rRNA gene, data on the microbial populations of the collected arthropods are presented. These findings suggest that arthropods may act as reservoirs and vectors of pathogenic microorganisms, potentially contributing to the transmission of HAIs by contaminating hospital surfaces, with the possibility of subsequent patient exposure.

## Methods

### Characterization of the surgical area

This study was conducted under an academic and research agreement between Universidad Libre Barranquilla and a Level III Healthcare Provider Institution (IPS) in Barranquilla, which provided the necessary resources and facilities for its execution. The IPS surgical service includes a dressing area for medical-surgical staff, lounges for nursing personnel and surgeons, a preoperative area, a recovery area, a handwashing area for surgical staff, operating rooms, and a postsurgical laundry and waste disposal area. These spaces are interconnected by doors, which serve as access control points for different personnel within the service. All areas were approved by the institution for sampling.

### Collection of specimens (Arthropods)

Arthropod collection was carried out in the aforementioned areas using entomological forceps, entomological nets, and manual aspirators as the primary collection methods. Additionally, active BG-Sentinel traps were deployed; however, no specimens were recovered from them. The collection process lasted 24 hours, divided into two 12-hour sessions on different days. Captured organisms were placed in sterile vials labeled with detailed collection data and subsequently transported to the laboratory for identification and processing.

### Taxonomic identification

In the entomology laboratory, the morphological characteristics of the arthropods were examined, and taxonomic identification was performed primarily at the family and genus levels using taxonomic keys. Upon sample reception, each arthropod underwent a surface wash using 1 mL of saline solution. The resulting wash solution was transferred into a sterile vial labeled with the corresponding collection data. Thus, each arthropod was stored in an individual sterile vial, accompanied by an additional vial containing the wash solution, both labeled identically. All samples were preserved at −20 °C and later transported to the molecular biology laboratory.

### Molecular analysis

The vials containing the specimens and their wash solutions were received at the Laboratorio de Investigaciones en Biomembranas of Universidad Libre, where molecular analyses were conducted. Genomic DNA (gDNA) extraction was performed using the QIAGEN DNeasy PowerLyzer PowerSoil Kit. Following extraction, DNA quantification was carried out by measuring absorbance at 260 nm using the NanoDrop™ 2000 spectrophotometer (Thermo Scientific™). The extracted gDNA was stored at −20 °C for microbial diversity experiments. For deep sequencing of the 16S ribosomal RNA gene, specifically the V3 and V4 variable regions, the following primers were used:

**Bakt_341F:** CCTACGGGNGGCWGCAG

**Bakt_805R:** GACTACHVGGGTATCTAATCC

Deep sequencing was performed using the Illumina MiSeq platform, generating paired-end reads of 300 bases each. Sequence reads were filtered using a quality threshold of Q30, with singletons and sequences shorter than 200 bases removed. Sequence quality control and classification analyses were conducted using the latest version of the MOTHUR platform.

## Results

### Insects collected in surgical areas

**Figure 1.**
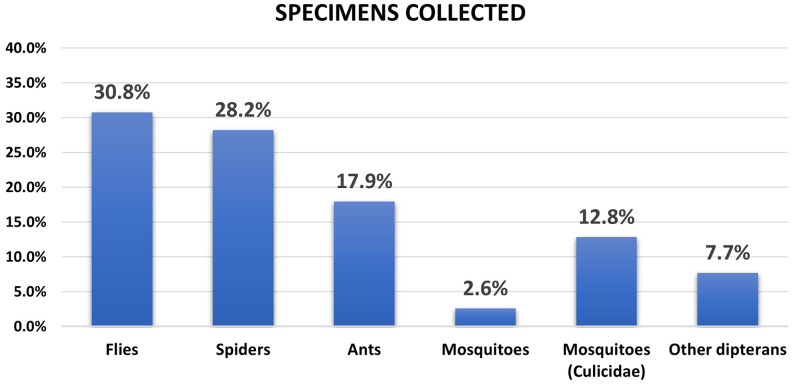
Proportion of individuals per morphotype collected in operating room areas of an IPS in Barranquilla, Colombia. The captured specimens were grouped into six insect categories based on their morphological characteristics.

Using the sampling techniques in operating room areas, a total of 30 samples were obtained. One of these was discarded due to deterioration, leaving 29 samples corresponding to 39 individuals (Figure 1). The parasitoid fly from the family Tachinidae accounted for the highest abundance, with 12 individuals representing 30.8% of the total collected, followed by red ants of the genus *Solenopsis* with seven individuals (17.9%). It is important to highlight that flies from the *Tachinidae* family are highly diverse with varied habits; however, most of these flies are parasitoids, and some species inhabit environments with decomposing organic matter, where they find other arthropods to parasitize. Adults feed on flower nectar and other plant-derived products. On the other hand, ants from the genus *Solenopsis* are important indicators of carbohydrate availability, primarily sugars derived from food residues.

### Characterization of the studied areas based on collected arthropods

Based on arthropod collection activities in operating room areas, the dressing room area showed the highest frequency of captures, contributing ten samples and 14 individuals, corresponding to 34% of the total samples. This was followed by the medical staff lounge, where seven samples were collected, with an equal number of individuals (17%) (Table 1). No insects were observed inside the operating rooms. Notably, among the evaluated areas, the location with the highest number of registered specimens was the farthest from the operating rooms, corresponding to the dressing room and food consumption area. It is likely that most of the insects found are attracted by odors and food residues, while other arthropods, such as spiders, are drawn to hunt these insects for sustenance. In the case of *Tachinidae* flies, their presence is likely due to their search for host insects to parasitize and lay their eggs. However, their presence is not strictly limited to this biological behavior.

**Table 1.** Summary of the distribution of the evaluated operating room areas and corresponding number of samples and individuals

Area	Number of samples	Number of specimens	Number of morphospecies
**Laundry**	5	9	4
**Lounge**	7	7	3
**Dressing room**	10	14	6
**Surgical hallway**	6	7	3
**Surgery stairs**	1	2	1
**Total**	29	39	17

### Microbiota analysis of insects found in surgical areas

To analyze the resident microbiota in the insects, 10 pools were formed with the 39 collected individuals, corresponding to the 10 identified morphospecies: *Tachinidae* family, *Solenopsis* genus, *Culicoides* genus, *Pholocidae* family, *Salticidae* family, *Theridion* genus, *Aedes* genus, *Psycodidae* family, *Sitotroga* genus, and *Musca domestica*. The extracted DNA samples were used for bacterial microbial diversity (metataxonomic) experiments, using the 16S rDNA molecular marker, variable regions V3–V4. The coverage analysis for the samples processed for microbial diversity showed a coverage value >99% (Table 2), indicating that the sampling was sufficient and had the appropriate characteristics for studying the diversity in the samples of interest.

**Table 2.** General results of alpha diversity analyses

Sample	Read count	Q30 (%)	nseqs	Coverage	Observed (Sobs)	Chao1	Shannon	Simpson
c16SINULB01	135,702	86.5	19 340	.99	46	46.5	2.3	.8
c16SINULB02	312,528	86.9	19 342	.99	144	189.3	2.9	.8
c16SINULB03	169,348	81.8	19 338	.99	112	114.3	2.5	.7
c16SINULB04	124,040	86.4	19 343	.99	75	80.0	2.3	.7
c16SINULB05	287,034	86.3	19 347	.99	218	279.1	2.9	.8
c16SINULB06	334,434	87.7	19 342	.99	86	112.0	2.3	.8
c16SINULB07	311,616	87.3	19 343	.99	57	57.3	2.1	.7
c16SINULB08	278,580	86.6	19 345	.99	136	159.8	1.3	.6
c16SINULB09	323,238	88.2	19 343	.99	45	45.5	2.3	.8
c16SINULB10	240,440	87.5	19 338	.99	119	123.5	2.6	.8

**Figure 2.**
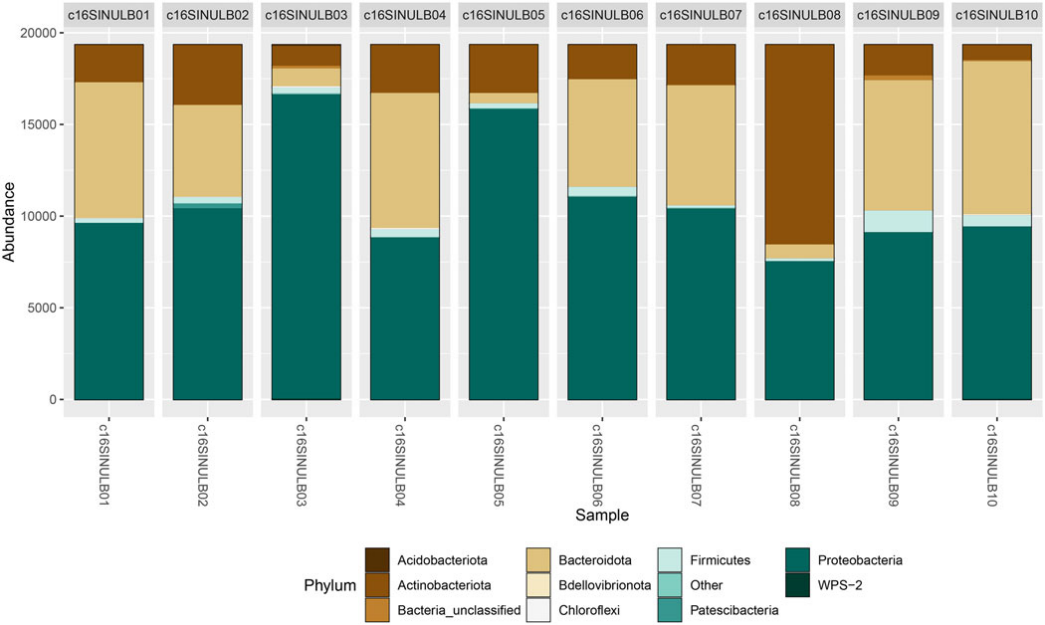
Taxonomic assignment at the phylum level. The graph shows the reads obtained for bacterial phyla.

Our data revealed that *Proteobacteria* was the predominant phylum (Figure 2). These findings are expected, given the broad diversity of bacterial species included in this taxon. *Proteobacteria* is the second-largest group of bacterial species, primarily composed of mesophilic Gram-negative bacteria with an affinity for neutral pH environments. This phylum includes a wide range of free-living microorganisms, which could be associated with the greater presence of species such as *E. coli* and certain species within the *Pseudomonadaceae* family, which are commonly found as free-living microorganisms in soil.^14^ Additionally, the presence of insects that predominantly feed on decomposing organic matter likely contributes to the presence of genetic segments from *Proteobacteria* species that thrive in such environments rich in readily assimilable carbon molecules.

**Figure 3.**
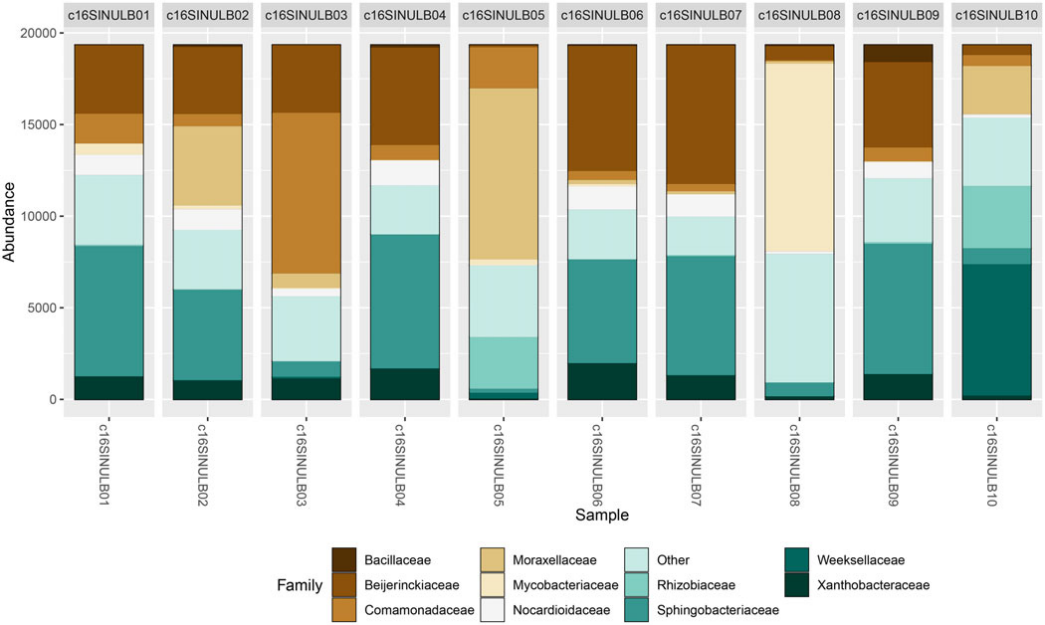
Taxonomic assignment at the family level. The graph shows the reads obtained for bacterial families.

For the analysis of bacterial sequences at the family level, we did not find a clear predominance of families of particular interest to human health. However, sequencing revealed the presence of bacterial families that include pathogenic species relevant to public health. Interestingly, the *Moraxellaceae* and *Mycobacteriaceae* families were particularly abundant in two of the 10 sequenced samples (Figure 3). Within the *Moraxellaceae* family, the presence of Gram-negative bacteria such as the *Acinetobacter* genus stands out, as it is associated with antibiotic-resistant nosocomial infections.^15,16^ On the other hand, the presence of the *Mycobacteriaceae* family strongly suggests the occurrence of non-tuberculous mycobacteria species, which predominantly infect patients undergoing cosmetic surgeries.^17–19^ Other families of interest that were detected and include species relevant to health were *Pseudomonadaceae* and *Alcaligenaceae*, which comprise bacterial species that cause antibiotic-resistant infections due to their ability to produce extended-spectrum beta-lactamases (ESBLs).^20,21^

### The bacterial genera identified are associated with potentially pathogenic bacteria

**Figure 4.**
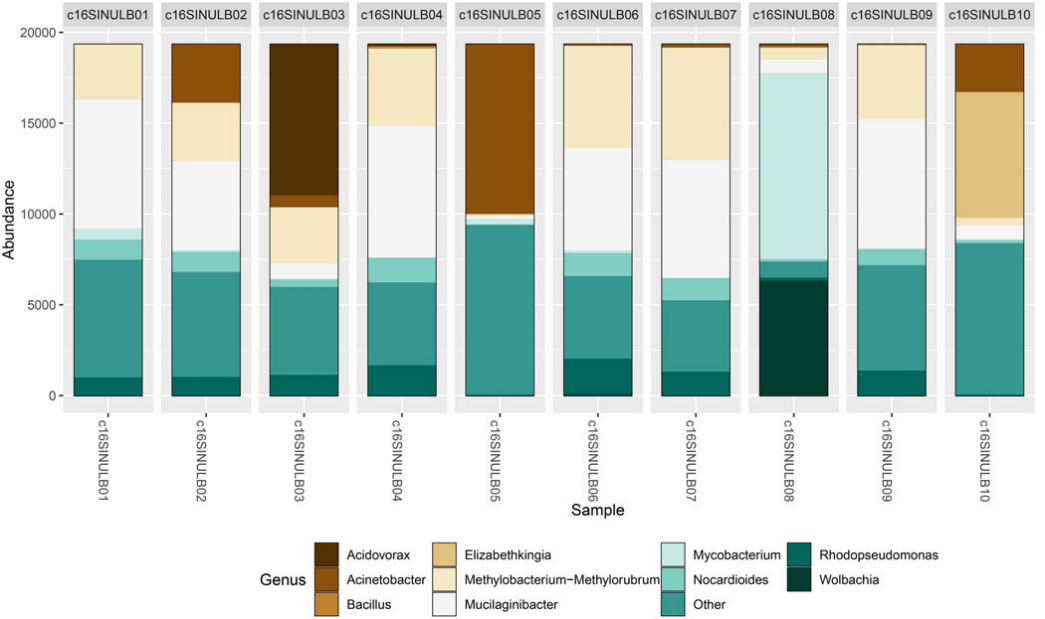
Taxonomic assignment at the genus level. The graph shows the reads obtained for bacterial genera.

Interestingly, when analyzing the sequences at a deeper taxonomic level, we identified the presence of potentially pathogenic genera such as *Acinetobacter* and *Mycobacterium* (Figure 4). Although the *Pseudomonas* genus was detected in very low proportions, its presence could suggest a risk of nosocomial infections. Other genera identified, such as *Bacillus*, were not considered particularly relevant due to their spore-producing ability, which allows them to spread widely in the environment and contaminate ventilated spaces. Although they have low clinical relevance in infections, *Bacillus cereus* has been responsible for significant nosocomial infection outbreaks that are difficult to manage.^22^ Genera particularly abundant in the mammalian intestine, such as *Escherichia* and *Shigella*, were detected in low proportions, suggesting a low presence in insects.^23,24^

## Discussion

Our study, through a descriptive observational approach, highlights the importance of insect control in hospital environments. Cleaning and sanitization in surgical areas and other critical zones of healthcare facilities are key strategies to prevent the proliferation of environmental and nosocomial pathogens. Although the population of arthropods identified in this study was small, it is essential to implement stricter control measures to avoid the presence of mechanical vectors that may contribute to the dissemination of pathogenic bacteria within the hospital complex.

The detection of bacterial genera with high pathogenicity and the ability to develop antibiotic resistance constitutes a warning about the relevance of these contaminating organisms, whose presence is more frequent in residential environments. Despite the toxicity of chemical agents used in hospital sanitation, we have identified small populations of arthropods that may persist in less controlled areas, such as washing zones and break rooms, where food is stored and consumed at certain times of the day.

This leads us to a key question in the study of nosocomial bacterial infections: what is the source of contamination? This question becomes particularly important when considering isolated bacterial species that do not have a close relationship with humans. Some rare infections are caused by free-living microorganisms that primarily inhabit soil and geographically isolated ecosystems. While the movement of patients and their relatives may facilitate their introduction into the hospital environment, sanitization procedures should almost eliminate the presence of microorganisms not typically found in urbanized areas.

Espíndola do Nascimento et al demonstrated the importance of the presence of ants in a hospital in Brazil^25^ as mechanical vectors of pathogenic bacteria. They showed the presence of Pseudomonas species. Although they were not the most abundant in our findings, our results confirmed their presence. In 2024, Frickmann et al showed the significance of ants in hospitals due to their ability to transport bacteria.^26^ While most studies focus on identifying microorganisms transmitted by cockroaches,^27^ few studies report the presence of pathogenic bacteria in insects of lesser impact on human health, such as ants, parasitoid flies, and domestic moths.^28^

Our study presents a new perspective in which mechanical vectors play a fundamental role in the life cycle of opportunistic bacteria capable of causing difficult-to-treat infections in hospitalized patients. This evidence underscores the need to strengthen arthropod control strategies in hospitals and further investigate their role in the epidemiology of nosocomial infections.

## Conclusion

The management and control of insect pests in hospital environments should be integrated as a routine biosafety practice. Our study provides evidence of the presence of multidrug-resistant bacteria associated with insects, supporting their active role in the dissemination of pathogens in hospitals. These findings highlight the need to strengthen pest prevention and control strategies to reduce the risk of nosocomial infections.

## References

[1] Serrano M , Barcenilla F , Limón E. Infección nosocomial en centros sanitarios de cuidados prolongados. Enfermedades Infecciosas y Microbiología Clínica. 2014;32:191–198. 24447921 10.1016/j.eimc.2013.11.007

[2] Abubakar U , Amir O , Rodríguez-Baño J , Healthcare-associated infections in Africa: a systematic review and meta-analysis of point prevalence studies. J Pharm Policy Pract. 2022;15:99. 36494700 10.1186/s40545-022-00500-5 PMC9733066

[3] Storr J , Twyman A , Zingg W , et al. Core components for effective infection prevention and control programmes: new WHO evidence-based recommendations. Antimicrob Resist Infect Control. 2017;6:6. 28078082 10.1186/s13756-016-0149-9 PMC5223492

[4] Delgado-Serrano J , Albarracin Ruiz MJ , Rangel-Vera JA , Galeano-Salazar E , Niño-vargas D , Wilches-Cuadros MA , et al. Perfil de resistencia antimicrobiana de aislamientos bacterianos en pacientes con infección urinaria de un centro de referencia en Bucaramanga. MedUNAB. 2020;23:405–422.

[5] Bolaño-Arenas J , Vásquez-Avendaño E , Márquez-Blanco N , Alvarino MAD Infecciones asociadas a la atención sanitaria y su relación con los insectos como vectores de transmisión en áreas quirúrgicas. Revista Colombiana de Entomología. 2023;49.

[6] Boyer PH , Koetsveld J , Zilliox L , et al. Assessment of Borrelia miyamotoi in febrile patients and ticks in Alsace, an endemic area for lyme borreliosis in France. Parasites Vectors. 2020;13:199. 32303256 10.1186/s13071-020-04071-9 PMC7165395

[7] Brinkmann A , Hekimoğlu O , Dinçer E , Hagedorn P , Nitsche A , Ergünay K. A cross-sectional screening by next-generation sequencing reveals Rickettsia, Coxiella, Francisella, Borrelia, Babesia, Theileria and Hemolivia species in ticks from Anatolia. Parasites Vectors. 2019;12:26. 30635006 10.1186/s13071-018-3277-7 PMC6329055

[8] Chadee DD , Le Maitre A. Ants: potential mechanical vectors of hospital infections in Trinidad. Trans R Soc Trop Med Hyg. 1990;84:297. 2389326 10.1016/0035-9203(90)90294-o

[9] Costa SBD , Pelli A , Carvalho GPD , et al. Formigas como vetores mecânicos de microorganismos no Hospital Escola da Universidade Federal do Triângulo Mineiro. Rev Soc Bras Med Trop. 2006;39:527–529. 17308696 10.1590/s0037-86822006000600003

[10] Daniel M , Srámová H , Absolonová V. Arthropods in a hospital and their potential significance in the epidemiology of hospital infections. Folia Parasitol (Praha). 1992;39:159–170. 1644363

[11] Fotedar R , Banerjee U , Singh S , Shriniwas, Verma, AK . The housefly (Musca domestica) as a carrier of pathogenic microorganisms in a hospital environment. J Hospi Infect 1992;20:209–215. 10.1016/0195-6701(92)90089-5 1348776

[12] Fotedar R , Shriniwas, Banerjee U , Samantray JC , Nayar E , Vermat A. Nosocomial infections: cockroaches as possible vectors of drug-resistant Klebsiella. J Hosp Infect 1991;18:155–159. 1678762 10.1016/0195-6701(91)90161-z

[13] López-Cerero L Papel del ambiente hospitalario y los equipamientos en la transmisión de las infecciones nosocomiales. Enferm Infecc Microbiol Clin. 2014;32:459–464. 24315300 10.1016/j.eimc.2013.10.004

[14] LaBauve AE , Wargo MJ. Growth and laboratory maintenance of *pseudomonas aeruginosa*. CP Microbiol. 2012;25. 10.1002/9780471729259.mc06e01s25 PMC4296558 22549165

[15] Gupta N , Gandham N , Jadhav S , Mishra RN. Isolation and identification of Acinetobacter species with special reference to antibiotic resistance. J Nat Sci Biol Med 2015;6:159–162. 25810655 10.4103/0976-9668.149116 PMC4367029

[16] Shi W , Wen D , Chen C et al. β-Lactamase production and antibiotic susceptibility pattern of Moraxella catarrhalis isolates collected from two county hospitals in China. BMC Microbiol. 2018;18:77. 30029595 10.1186/s12866-018-1217-5 PMC6054730

[17] Leto Barone AA , Grzelak MJ , Frost C , et al. Atypical mycobacterial infections after plastic surgery procedures abroad: a multidisciplinary algorithm for diagnosis and treatment. Ann Plast Surg 2020;84:257–262. 31688120 10.1097/SAP.0000000000002061

[18] Safe IP , Macedo V , Marcelo W , et al. Nontuberculous mycobacterial infections after aesthetic procedures: comparison of clinical features and treatment. J Clin Aesthet Dermatol 2021;14:46–49. PMC8021410 33841617

[19] Shaikh U , Hussain A. 50599 reviewing the risks of medical tourism: atypical mycobacterial infections associated with cosmetic procedures abroad. J m Acad Dermatol. 2024;91:AB301.

[20] Huang C. Extensively drug-resistant Alcaligenes faecalis infection. BMC Infect Dis. 2020;20:833. 33176714 10.1186/s12879-020-05557-8 PMC7659064

[21] Puah SM , Puthucheary S , Chua KH. First report of extended-spectrum β-Lactamases TEM-116 and OXA-10 in clinical isolates of alcaligenes species from Kuala Lumpur, Malaysia. Jpn J Infect Dis. 2019;72:266–269. 30918144 10.7883/yoken.JJID.2018.031

[22] Glasset B , Herbin S , Granier SA , et al. Bacillus cereus, a serious cause of nosocomial infections: epidemiologic and genetic survey. Koehler TM, ed.PLoS ONE. 2018;13:e0194346. 29791442 10.1371/journal.pone.0194346 PMC5966241

[23] Müller A , Seinige D , Grabowski NT , Ahlfeld B , Yue M , Kehrenberg C. Characterization of Escherichia coli from edible insect species: detection of shiga toxin-producing isolate. Foods. 2021;10:2552. 34828833 10.3390/foods10112552 PMC8618678

[24] Van Looveren N , IJdema F , Van Der Heijden N , Van Der Borght M , Vandeweyer D. Microbial dynamics and vertical transmission of Escherichia coli across consecutive life stages of the black soldier fly (Hermetia illucens). Anim microbiome. 2024;6:29. 38797818 10.1186/s42523-024-00317-4 PMC11129375

[25] Do Nascimento LE , Amaral RR , Ferreira RMDA , et al. Ants (Hymenoptera: Formicidae) as potential mechanical vectors of pathogenic bacteria in a public hospital in the Eastern Amazon, Brazil. J Med Entomol. 2020;57:1619–1626. 32368780 10.1093/jme/tjaa062

[26] Frickmann H , Hurtig S , Greine AR , et al. Risk assessment of the mechanical spread of bacterial pathogens due to lasius neglectus ants infesting a tertiary hospital. J Hosp Infect 2024;150:83–90. 38823645 10.1016/j.jhin.2024.04.026

[27] Kakooza S , Ssajjakambwe P , Nalubega R , et al. Cockroaches as reservoirs, vectors, and potential sentinels of multidrug-resistant bacteria in Ugandan communities: a retrospective analysis. Glob Health Epidemiol Genom. 2025;2025: 5940509. 39872438 10.1155/ghe3/5940509 PMC11769582

[28] Boiocchi F , Davies MP , Hilton AC. An examination of flying insects in seven hospitals in the United Kingdom and carriage of bacteria by true flies (Diptera: Calliphoridae, Dolichopodidae, Fanniidae, Muscidae, Phoridae, Psychodidae, Sphaeroceridae). J Med Entomol. 2019;56:1684–1697. 31225584 10.1093/jme/tjz086

